# Profiling Italian cat and dog owners’ perceptions of pet food quality traits

**DOI:** 10.1186/s12917-020-02357-9

**Published:** 2020-05-11

**Authors:** Marica Vinassa, Diana Vergnano, Emanuela Valle, Marzia Giribaldi, Joana Nery, Liviana Prola, Domenico Bergero, Achille Schiavone

**Affiliations:** 1 Department of Veterinary Sciences, University of Turin, Largo Paolo Braccini 2, 10095 Grugliasco, TO, Italy; 2 CNR, University of Turin, Largo Paolo Braccini 2, 10095 Grugliasco, TO, Italy; 3 Research Centre for Engineering and Agro-Food Processing, CREA, Strada delle cacce, 73, 10135 Turin, Italy

**Keywords:** Pet food, Quality indicators, Survey, Perception of pet food

## Abstract

**Background:**

Over recent years, pet owners have started to demonstrate increased sensitivity toward their companion animals, which includes an increase in the attention paid towards their nutrition, seen as a way of safeguarding their pets’ welfare. The aim of this study was to identify how pet food quality traits are perceived as being the most important by dog and cat owners. To this end, a survey of dog and cat owners was conducted by means of a questionnaire distributed in pet stores and trade fairs throughout Italy.

**Results:**

A total of 935 surveys were collected; 61.8% of which were compiled by female pet owners. The respondents were relatively homogeneously distributed between cat (30.8%), dog (39.4%), and cat and dog (29.8%) owners. A quarter of the owners (25.5%) reported to have asked their veterinarian for advice on which pet food to buy, and almost a third (30.4%) trusted the advice posted on the web sites of well-known brands. “Contains natural ingredients” was the characteristic that obtained the highest mean score (4.3 out of 5).

Elderly owners (> 65 years) placed most importance on whether a product had a high price, and least on feed appearance, animal satisfaction, and stool quality. Young owners (< 35y) paid most attention to the stool quality, the percentage of protein in the feed, and the presence of recyclable packaging, and least attention to feed appearance, smell, and animal satisfaction. Feed appearance, smell, a higher cost, and certain label indications (protein content, presence of fresh meat, grain free) were mostly important among the buyers of wet pet food. Some specific differences also emerged between dog, cat, and dog and cat owners.

**Conclusions:**

In this survey of Italian pet food buyers, the presence of “natural” ingredients was considered to be the most important indicator of pet food quality, whereas characterized by a high price was considered least important. The data obtained from this survey could be used to help pet food companies identify which pet food quality traits are perceived as important by dog and cat owners.

## Background

Around 39% of the population in Italy own at least one cat or dog [1], and over the last 10 years the dog and cat feed market has shown significant positive growth [1]. Furthermore, pet owners are becoming more sensitive toward pet care issues [1], and nutrition is seen as a way of safeguarding their animals’ welfare. However, different pet owners consider different and specific criteria they expect the pet food to fulfill, which inevitably determine the diet choices they make for their pets. Nowadays, pet owners are more aware about the importance of the quality of their pets’ feeds and the ingredients they contain. Furthermore, it is known that different social and cultural factors can influence the decision-making processes underlying pet food purchasing, the same that govern consumer choices in relation to their own food purchasing habits. According to Michel et al. [2], wholesomeness, perceived ingredient safety, and perceptions about nutritional value are the major features that influence pet owner choices, together with their sources of information about pet nutrition. In fact, pet food buyers have access to numerous sources of information about pet nutrition (veterinarians, the internet, animal trainers, pet shop employees, books on pet nutrition, pet nutrition company websites, other pet owners, etc.), even though this information may not always derive from reliable sources [3]. Some feed features are perceived as positive and linked to health benefits, such as “organic” or “grain free”, whereas certain ingredients, such as wheat and corn, sometimes may be considered as negative features because they have been associated with the perception of being potentially harmful [4]. Pet food producers take this information into account, and promote their products using claims such as “cruelty-free”, “organic”, and “natural” [5, 6]. The quality of the complete commercial feed is cited as a contributing factor for longer and healthier lives in pets [7]. Furthermore, their use is widespread in developed countries, as it is in Italy [1], even though interest in alternative feeding strategies are also on the rise [8–11].

This growing trend to pay more attention to specific pet food characteristics is affecting consumers‘purchasing choices [6, 12], whose decisions are now influenced more by the perceived quality than by price [6, 13]. According to Landes et al. [14], dog and cat owners prefer to spend more money to buy premium feeds rather than pet accessories (e.g. toys, collars, etc.). The social and cultural factors that influence pet owners’ own eating habits also influence the decision-making processes underlying pet food purchasing and pet feeding practices [15].

The aim of the study was to identify which pet food quality traits are perceived as being the most important by dog and cat owners. To this end, a survey was developed to investigate the relevant habits and attitudes of dog and cat owners when choosing and purchasing their pets’ food.

## Results

A total of 972 questionnaires were distributed during the observation period; following the elimination of incomplete questionnaires, 935 were left for analysis.

The socio-demographic characteristics of the surveyed pet owners are reported in Table 1. Sixty-one point 8 % were women. The majority of questionnaires were completed by people in work and with a medium-high level of education. Amongst half (49.5%) the tested population was resident in the regions that make up Northern Italy (Piedmont, Valle d’Aosta, Liguria, Lombardy, Trentino Alto Adige, Veneto, Friuli-Venezia Giulia, and Emilia-Romagna), 34.4% were living in Central Italy (Tuscany, Umbria, Marche, and Lazio), and 16.1% were from the South (Abruzzo, Molise, Campania, Puglia, Basilicata, and Calabria) or the Islands (Sicily and Sardinia). The number of interviews conducted with cat (30.8%), dog (39.4%) and cat and dog (29.8%) owners was quite evenly distributed.

**Table 1:** Socio-demographic characteristics of the surveyed pet owner population

Characteristics	n° and % of valid responses
**Gender**	(*n* = 935)
Women	61.8
Men	38.2
**Age**	(*n* = 929)
18–34 years	31.5
35–50 years	38.6
51–64 years	22.1
> 64 years	7.8
**Geographical area of residence**	(*n* = 932)
Northwest Italy	29.0
Northeast Italy	20.5
Central Italy	34.4
Southern Italy and the Islands	16.1
**Educational level**	(*n* = 893)
Primary / secondary school	14.4
High school / professional qualification	58.1
Degree / Master	27.4
**Occupation**	(*n* = 931)
Student	15.7
Housewife	8.8
Retired	8.6
Worker	61.5
Unemployed	3.1
Other	2.3
**Animal(s) owned (dogs and/or cats)**	(n = 932)
Dogs	39.4
Cats	30.8
Dogs and cats	29.8

Some questions were used to profile the purchasing habits of the participants (Table 2). The majority (65.3%) of the sampled population bought both dry and wet pet food, whereas about 10% purchased wet pet food only. The preferred marketing channel of the tested population was a pet store (63.3%). About one quarter (25.5%) of the interviewees had asked for advice from their veterinary about which pet food to purchase, and almost one-third (30.4%) relied on the information provided by major brands on their websites.

**Table 2:** Purchasing habits of the surveyed pet owners

Type of pet food purchased (***n*** = 914)	%	Preferred marketing channel for the pet food (***n*** = 915)	%	Prime source of nutritional advice used (***n*** = 931)	%
Dry	24.7	Supermarket	15.8	Friends and relatives	13.0
Wet	10.0	Pet store	63.3	Online blog	9.8
Dry & wet	65.3	Online	6.6	Online website	30.4
		More than one	14.3	Veterinarian	25.5
				Other	6.7
				More than one	14.7

Table 3 reports the percentage of responses awarding each score value of the 1–5 Likert scale (where 1 = not important, and 5 = fundamental) in relation to each quality characteristic assessed and the average score considering the whole population. The claim “contains natural ingredients” had the highest average score considering the responses from all interviewees, whereas the characteristic “higher price than others” had the lowest average score, indicating that, in the surveyed population, purchasing decisions were not made on the basis of the product having a high price.

**Table 3:** Average relevance score of the surveyed quality characteristics of the chosen pet food

Characteristics	Score (% for each category)					Average score (***n*** = 935)
	1	2	3	4	5	
Contains natural ingredients	0.4	3.7	15.3	29.7	50.9	4.3
Location of pet food production facilities clearly labeled	0.9	3.8	16.8	31.0	47.5	4.2
Comprehension of the label	1.0	4.8	19.0	27.3	47.9	4.2
Pet’s preference (i.e. palatability)	0.3	2.7	18.5	32.5	46.0	4.2
Normal stool appearance	0.7	2.8	18.8	33.4	44.3	4.2
Contains fresh meat	1.5	6.0	21.5	27.1	43.9	4.1
Cruelty free	3.3	6.9	23.1	22.6	44.1	4.0
Produce shiny coat	1.0	5.0	22.7	35.5	35.8	4.0
Meat as the main ingredient	2.2	7.3	23.2	26.7	40.6	4.0
Good food smell	3.3	11.6	26.5	33.6	25.1	3.7
High protein content	1.3	8.5	28.7	37.9	23.5	3.7
Food appearance	4.3	10.8	29.0	31.5	24.4	3.6
Grain free	6.0	19.4	32.2	24.6	17.8	3.3
Recyclable packaging	13.9	18.3	24.5	22.0	21.3	3.2
Known brand	12.0	20.3	28.8	25.3	13.5	3.1
Higher price than other products	24.1	27.6	26.9	13.7	7.7	2.5

(1 = not important, 5 = fundamental)

### Correlation analysis

The correlation analysis showed that less than 50% of correlations were relevant (Table 4). The location of the pet food production facilities correlated with the presence of specific information on the label (comprehension of label R = 0.660; contains natural ingredients R = 0.584; cruelty-free R = 0.564). The importance placed on whether the food gave their pet a shiny coat correlated with the importance placed on stool appearance (R = 0.579); and the importance placed on the pet’s preferences (i.e. palatability) correlated with the importance placed on both shiny coat and stool appearance (R = 0.591 and R = 0.529, respectively). Consumer preference for the food being a well-known brand correlated with importance attributed to a high price (R = 0.616). A correlation was also observed between the importance of the food’s smell and its appearance (R = 0.761).

**Table 4:** Correlation coefficients between quality characteristics scored by the surveyed population

	Preference	Shiny coat	Stool	Food smell	Food appearance	Location of pet food production facilities	Cruelty-free	Comprehensive label	Natural ingredients	Meat main ingredient	Fresh meat	Total protein %	Grain free	Well-known brand	High price
**Recyclable**	0.302	0.297	0.324	0.256	0.228	0.359	0.428	0.414	0.373	0.209	0.209	0.233	0.430	0.305	0.416
**High price**	0.103 ns	0.218	0.101 ns	0.372	0.411	0.159	0.205	0.183	0.190	0.260	0.122 ns	0.228	0.447	**0.616**	
**Well-known brand**	0.168	0.177	0.085 ns	0.328	0.367	0.111 ns	0.139 ns	0.126	0.148	0.227	0.194	0.300	0.365		
**Grain free**	0.256	0.372	0.311	0.379	0.382	0.372	0.414	0.371	0.396	0.373	0.304	0.396			
**High protein %**	0.211	0.205	0.249	0.181	0.247	0.331	0.175	0.326	0.345	0.402	**0.511**				
**Fresh meat**	0.237	0.184	0.302	0.197	0.233	0.398	0.263	0.390	0.480	**0.649**					
**Meat main ingredient**	0.205	0.250	0.301	0.271	0.285	0.378	0.310	0.435	**0.521**						
**Natural ingredients**	0.310	0.284	0.384	0.216	0.219	0.584	0.508	**0.670**							
**Comprehensive label**	0.341	0.344	0.462	0.241	0.262	**0.660**	**0.643**								
**Cruelty-free**	0.361	0.396	0.459	0.339	0.352	0.564									
**Location of pet food** **production facilities**	0.331	0.377	0.494	0.305	0.338										
**Food appearance**	0.373	0.459	0.338	**0.761**											
**Food smell**	0.426	**0.524**	0.403												
**Stool appearance**	**0.529**	**0.579**													
**Shiny coat**	**0.591**														

ns: non significant correlation. Bold values are considered to be relevantly correlated (> 0.5)

### Multivariate correspondence analysis

Multivariate correspondence analyses were performed in order to underline any relevant associations between specific population segments and important factors in the decision-making process of pet food purchasing. The surveyed population was segmented according to age, educational level, occupation, geographical provenance, the type of pet food purchased, and the animal owned (dog or cat or both).

**Fig. 1:**
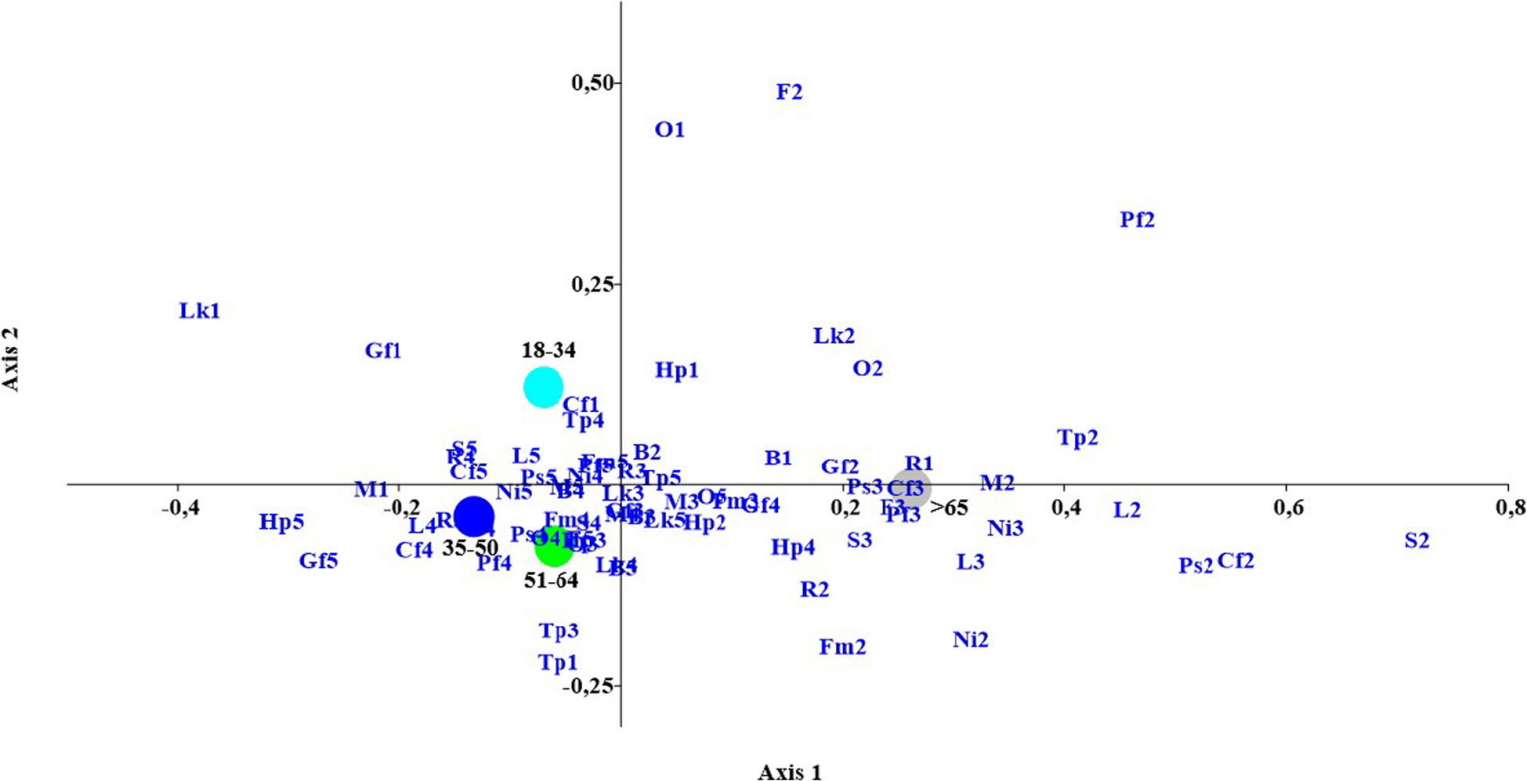
Results from multivariate correspondence analysis of the segmentation as per age class. *Legends*: *Pf1-Pf5*: pet preference (i.e. palatability); *F1-F5*: produces shiny coat; *S1-S5:* normal stool appearance; *O1-O5*: food smell; *Lk1-Lk5*: feed appearance; *Ps1-Ps5*: manufacturing facilities location; *Cf1-Cf5*: cruelty-free; *L1-L5*: label comprehension; *Ni1-Ni5*: presence of natural ingredients; *M1-M5*: meat as main ingredient; *Fm1-Fm5*: contained fresh meat; *Tp1-Tp5:* high protein content; *Gf1-Gf5*: grain free; *B1-B5*: well-known brand; *Hp1-Hp5*: higher price than others; *R1-R5:* recyclable packaging. Age classes: 18–34 y; 35–50 y; 51–64 y; > 64 y

The results of segmentation according to age category are shown in Fig. 1. Elderly respondents (> 65 y) reported a high price to be important to them when choosing pet food, whereas they attributed least importance to the use of recyclable packaging and the presence of the “cruelty-free” claim. Relatively little importance was also attributed by elderly people to certain aspects of label information, such as label comprehension, location of the pet food production facilities, and the presence of “natural” ingredients. Moreover stool quality was not considered as important by these respondents. On the other hand, young owners (< 35 y) placed most relevance on the appearance of the stools, a high percentage of proteins in the pet food, and to the presence of recyclable packaging. Furthermore, pet food appearance and smell were less relevant as was consideration of their pet’s preference. The “cruelty-free” and the “grain-free” claims received on average higher scores for the population aged 35 to 50, who also attributed a lot of importance on whether the pet food had a higher price than other products.

**Fig. 2:**
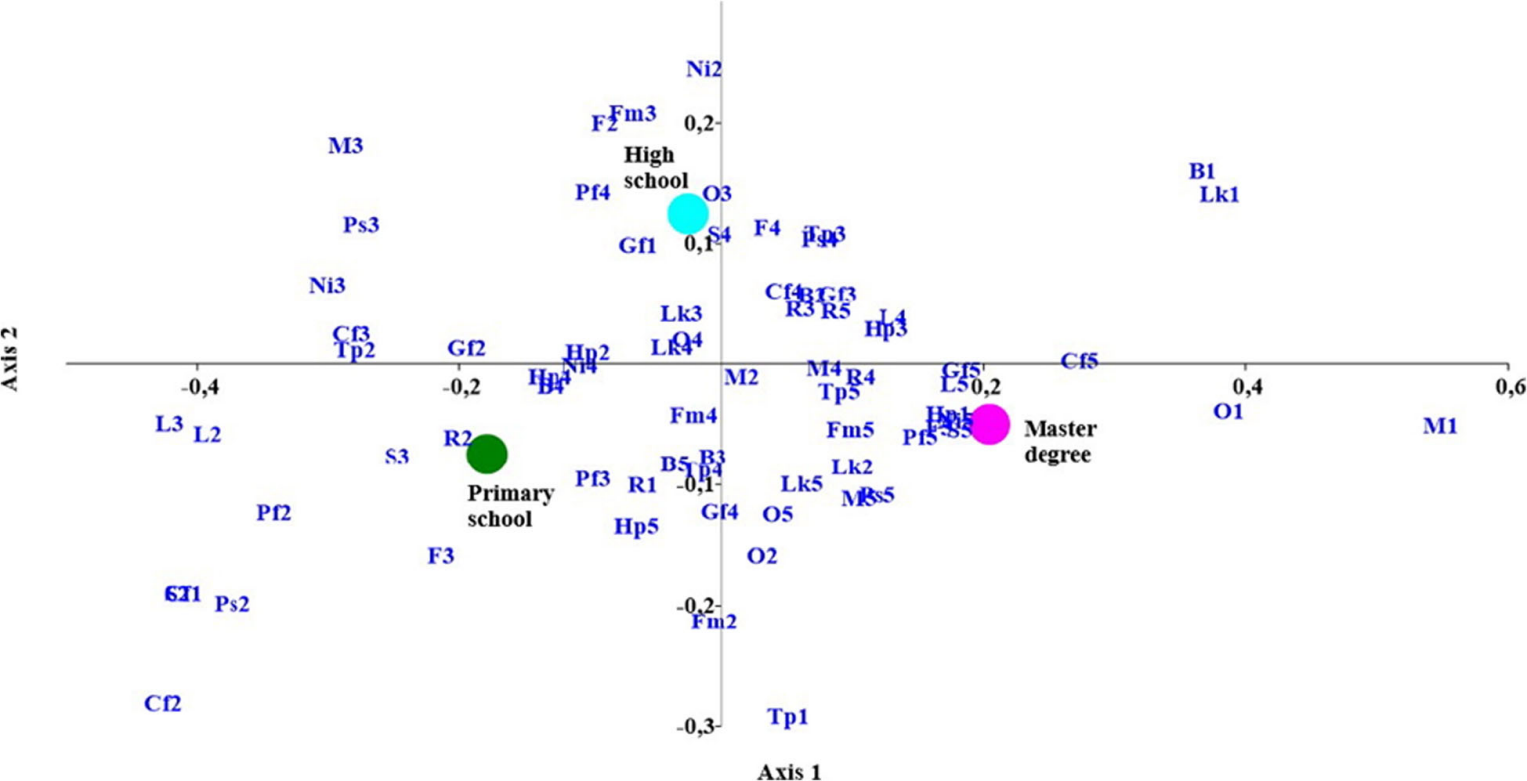
Results from multivariate correspondence analysis of the segmentation as per educational level. *Legends: Pf1-Pf5*: pet preference (i.e. palatability); *F1-F5*: produces a shiny coat; *S1-S5:* normal stool appearance; *O1-O5*: food smell; *Lk1-Lk5*: feed appearance; *Ps1-Ps5*: manufacturing facilities location; *Cf1-Cf5*: cruelty-free; *L1-L5*: label comprehension; *Ni1-Ni5*: presence of natural ingredients; *M1-M5*: meat as main ingredient; *Fm1-Fm5*: contains fresh meat; *Tp1-Tp5:* high protein content; *Gf1-Gf5*: grain free; *B1-B5*: well-known brand; *Hp1-Hp5*: higher price than others; *R1-R5:* recyclable packaging. Master’s degree: degree/specialization; High school: high school/professional qualification; Primary school: primary/secondary school

The results of the education level segmentation are given in Fig. 2. Pet owners with a higher educational level (degree/master) claimed to attribute little importance to the smell and appearance of the pet food, a high price, and to whether the brand is well-known, but they declared to attribute a high level of importance to the appearance of their pet’s stools and coat. Label comprehension, the location of production, the presence of natural ingredients, and grain-free and cruelty-free claims all received positive scores for this population segment. Unlike the respondents with a degree, the segment of the population with only a primary-school level of education considered certain aspects of the label information (production site, protein percentage, and label comprehension) as less relevant. The presence of a cruelty-free claim did not constitute a decisional factor for this population segment. A healthy stool and coat appearance was highly relevant for the interviewees with high school or professional qualifications, together with the presence of recyclable packaging.

Those working from home and housewives paid more attention to cruelty-free and grain-free claims. Label comprehension, feed smell and appearance, pet preferences, coat quality, and stool appearance were all rated by students as being of little importance. Given that our findings were based on an unbalanced number of housewives and students with respect to workers, the results from these analyses should be treated with considerable caution.

**Fig. 3:**
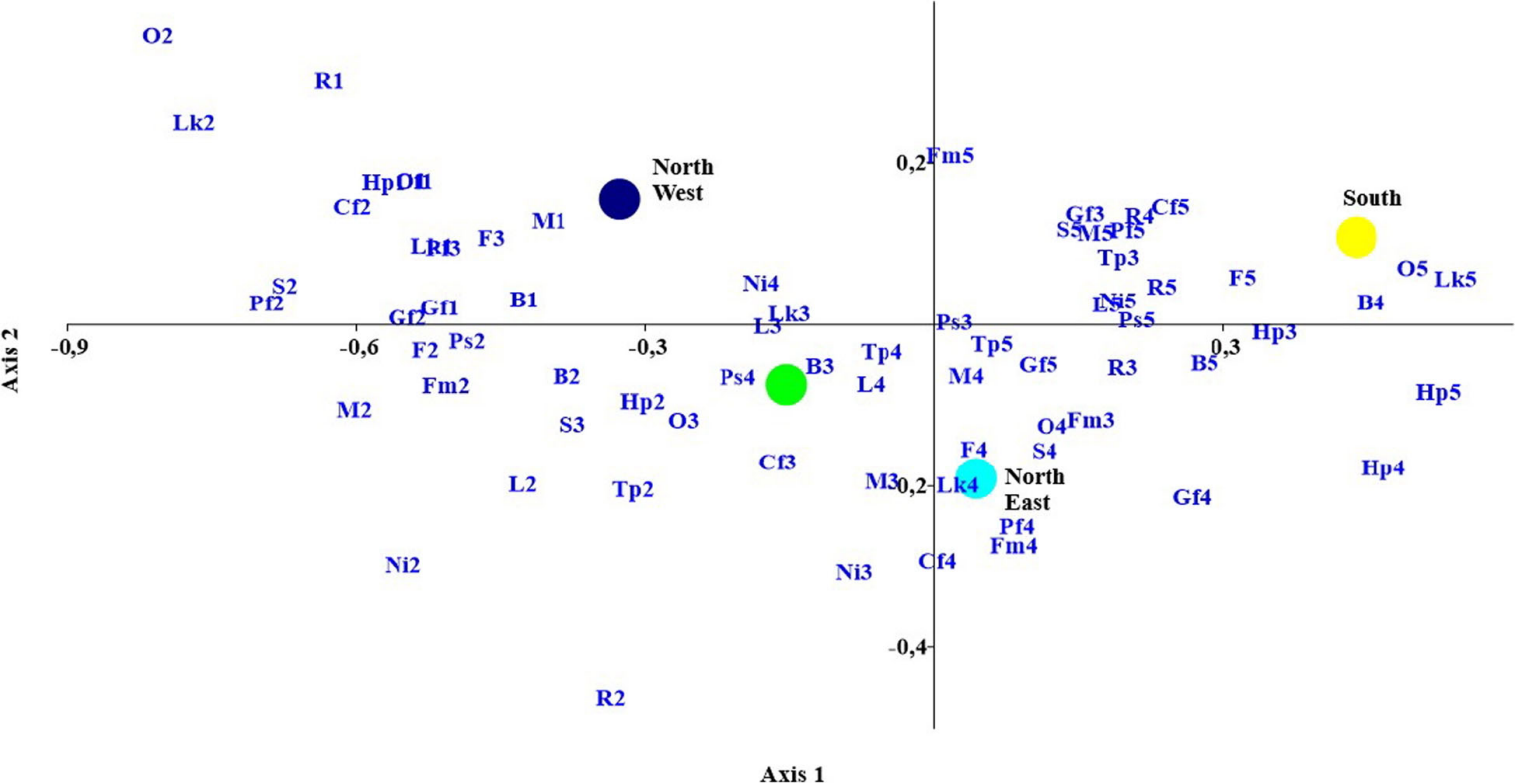
Results from multivariate correspondence analysis of the segmentation according to the macroscopic regions of the Italian peninsula. *Legends: Pf1-Pf5*: pet preference (i.e. palatability); *F1-F5*: produces a shiny coat; *S1-S5:* normal stool appearance; *O1-O5*: food smell; *Lk1-Lk5*: feed appearance; *Ps1-Ps5*: manufacturing facilities location; *Cf1-Cf5*: cruelty-free; *L1-L5*: label comprehension; *Ni1-Ni5*: presence of natural ingredients; *M1-M5*: meat as main ingredient; *Fm1-Fm5*: contains fresh meat; *Tp1-Tp5:* high protein content; *Gf1-Gf5*: grain free; *B1-B5*: well-known brand; *Hp1-Hp5*: higher price than others; *R1-R5:* recyclable packaging. North West: Northwest Italy (Piedmont, Valle d’Aosta, Liguria, Lombardy); North East: Northeast Italy (Trentino Alto Adige, Veneto, Friuli-Venezia Giulia, Emilia-Romagna); Center: Central Italy (Tuscany, Umbria, Marche, Lazio); South: Southern Italy (Abruzzo, Molise, Campania, Puglia, Basilicata, Calabria) and islands (Sicily, Sardinia)

The results from the segmentation according to the macroscopic regions of the Italian peninsula are reported in Fig. 3. It should be noted that the scores assigned by respondents in Southern Italy and the islands were always higher than those for the rest of Italy, whereas lower scores were selected in Northwest Italy. The mean score assigned to the importance of a high price was approximately 50% higher in Southern Italy compared with Northwest Italy.

The appearance, smell, higher cost, and some label indications (high protein content, meat as the main ingredient, grain free) were scored as being more relevant by wet pet food buyers. Cat owners attributed more importance to the appearance and smell of the pet food, but less to recyclable packaging, whereas dog owners focused more on the presence of meat as the main ingredient and on a healthy stool appearance.

## Discussion

Even though the survey was only administered in pet stores and trade fairs, thereby neglecting some pet food sectors (i.e. e-commerce, hypermarkets, supermarkets and discount stores), it was possible to highlight the main quality indicators Italian pet owners bear in mind when they choose their pet food. In this study, the pet owners were divided according to whether they owned a dog, a cat or both (39.4, 30.8, and 29.8%, respectively). These data corroborate the national report [1], a higher percentage of dog owners (27.1%) than cat owners (18.3%) have been found in Italian citizen. Of all respondents, 61.8% were women, confirming the trend revealed in a 2019 Italian study [16].

Our survey data reveal a higher incidence of pet stores as the “preferred shopping channel” (63.3%), but our data should only be considered as partial since the survey was conducted in pet stores and trade fairs. In 2019, Assalco-Zoomark [1] reported that the majority of pet food was bought in supermarkets (63.6% of all purchases, considering hypermarkets, supermarkets and discount stores), whereas little over a quarter was bought in pet stores (26.3%).

When assessing the quality of pet food, the Italian buyers in this study considered the presence of “natural” ingredients as the most important aspect (average score: 4.3). This may reflect the current trends also observed in relation to human nutrition, where demand is increasing for a more “natural” diet [17]. Thus, pet food is becoming more “humanized”, and so follows human food preferences and purchasing habits. The feed types chosen for pets are likely to reflect the relationship that has developed between the person and their animals. It could even be considered as symbolic of the pet’s inclusion into the owner’s family and reflect the pet owner’s culture or ideology. In fact, a recent study that investigated the relationship between children and household pets, 70.7% of the interviewed families considered the family pet as the child’s playmate [18]. Furthermore, some pet owners believe that choosing “natural” ingredients positively contributes to the health of their pets [15].

Other characteristics rated by the sample population as being important were: the location of the pet food production facilities (average score: 4.2), and the information provided on the label and its comprehension (average score: 4.2). The correlation analysis also revealed a link between these two aspects.

Another current trend in pet food marketing is the development of “grain free” products. According to Laflamme et al. [19], cereals in pet food may give rise to food adverse reactions. However, according to our survey, this characteristic was not deemed a major priority by the Italian pet owners interviewed. The average score of this feature was one of the lowest at 3.3. Other low-scoring characteristics were the presence of recyclable packaging (average score: 3.2) and the importance of a brand being well-known (average score: 3.1). According to Italian buyers, when considering a range of products (i.e. products with similar characteristics), a higher price (average score: 2.5) was the least important parameter.

Around a quarter of the interviewees (25.5%) asked their veterinarians for advice about the choice of pet food, suggesting the important role of veterinarians in the decision-making process of pet food purchasing. This result confirms the findings of a previous study [20] in which veterinarians were the most frequent source used to obtain information about pet nutrition. However, nearly a third of the interviewees (30.4%) reported to trust the details provided by pet food companies on their respective websites, suggesting that the pet food industry also plays an important role in the provision of information to consumers. In a study conducted in the United States and in Australia [20] on the attitudes of owners toward pet food, it was shown that a significant proportion of pet owners (15.8% of dog owners and 16.9% of cat owners) used the internet and other media as their primary sources of information.

The terminology “cruelty-free” is another aspect that should be taken into consideration, even though there are still some concerns about its definition. When talking about ingredients in cosmetic products, the United States Food and Drug Administration [21] considers “cruelty-free” products to infer that they have not been tested on animals. The tendency of pet owners to buy feeds that have not been tested on laboratory animals, commonly referred to as “cruelty-free” feed, is spreading in Italy. This aspect was perceived as being very important to the Italian interviewees (average score: 4.0). It is an aspect that the media and public opinions are focusing on; however, the term is not yet regulated by any specific legislation. Updating the legislation in reference to this topic therefore poses an important issue and could be a possible perspective for the Italian and European legislative bodies.

Other concerns revealed as important pertain to the comprehension of the label, shown to be one of the factors most important to the interviewees (average score: 4.2). However, it is important that the label should not mislead with regard to the feed’s use consumers, as clearly stated in Reg. (EC) No. 767/2009 on the placing of feed on the market and their use, as pointed out by FEDIAF (European Pet Food Industry Federation), which developed the Code of Good Labeling Practices [22]. Although Reg. (EC) No. 767/2009, which addresses on placing feeds onto the market and their use within the European Community, already includes general requirements for labelling. It could be a further step forward if the general requirements for labelling would be implemented for specific aspects, especially claims.

A distinction between two large categories of pet owners emerged from the correlation analysis. Interviewees that placed great importance on pet palatability preferences also paid attention to the appearance of their animal’s coat and stools. On the other hand, the interviewees interested in well-known brands also reported to pay attention to the price and external characteristics of the feeds (such as its appearance and the smell).

This study also revealed differences in how pet food quality is perceived that depended on the age of the interviewees. For example, elderly people did not tend to consider the use of recyclable packaging as important, which was deemed important to young pet owners (< 35 y), which may reflect a greater level of awareness of environmental impacts in this age group. Furthermore, of all the items considered, elderly interviewees placed most importance on whether a product cost more than other products, whereas they paid least attention to label information; on the other hand, the young interviewees (< 35 y) were most interested in the information written on the label. According to Mascarello et al. [23], age also influences the evaluation of the perceived quality of human food. It was interesting that the elderly people in our study seemed to behave in the same way as those in the human study by Mascarello et al. [23], in which they place less importance on nutritional aspects and more importance on marketing aspects. In that study [23], it was found that elderly people were most interested in buying certified and local food products.

The present study also reveals the respondents’ educational level to be associated with which pet food traits they perceive as being the most important. Similarly, in a human study, it was also reported that differences in the educational level of mothers were linked to differences in the eating habits of their children (the consumption of soft drinks, sweets, fruit and vegetables) [24].

When the data were segmented according to pet species, it resulted that cat owners were more interested in the features characterizing the pet food’s external appearance. In fact, cats are notoriously fussy when it comes to what they will eat, and cat owners know that pet food palatability is strongly influenced by both the smell and appearance (i.e. texture), as well as feed taste. On the contrary, dog owners express more interest on the nutritional composition of the feed, mainly on the quantity of protein, perhaps because of the common opinion that a dog’s diet should reflect the dog’s evolution from the ancestral wolf. A healthy stool appearance scored high among dog owners. Indeed, stool consistency problems are common in dogs, especially large dogs [25]. However, further information on the size of the respondents’ dogs would be necessary to understand whether a correlation existed between dog size and the importance attributed to stool consistency when purchasing dog food.

## Conclusion

To the best of our knowledge, this study provides the first data on the decision-making processes of Italian pet owners when purchasing commercial pet food. The feature rated by the Italian pet food buyers as being the most important quality indicator was the presence of “natural” ingredients, whereas least importance was attributed to whether products cost more others. Furthermore, our results highlight that the interviewees preferred to ask their veterinarians for advice on pet food or to consult the websites of specific brands, which were rated almost equally. Of the investigated parameters rated as most important to pet owners, some were linked to indicators of animal welfare (such as a shiny coat, normal stool appearance, and palatability). Some pet food quality parameters tended to be perceived differently depending on whether the respondent was a dog or cat owner. Furthermore, the pet owner decision-making process was strongly influenced by their level of education.

In conclusion, the obtained data here may be helpful to identify the factors that influence the perceptions of dog and cat owners regarding the quality of animal feed products.

## Methods

The survey (see Additional file 1 to view the English version) was designed to investigate the relevant habits and attitudes of dog and cat owners when choosing and purchasing pet food.

No approval by an institutional review board was required because enrollment was on a voluntary basis and the participants consented to anonymous data collection. In addition, the questionnaires were blinded prior to statistical analysis, which was performed independently by a specialist on a database containing summarized data.

### Respondents

The survey was designed and administered by trained staff over a period of 9 months, from March to November, 2018.

People were contacted directly by representatives promoting the survey in pet stores and trade fairs across Italy. The survey participants filled out the paper-format questionnaire and immediately returned it to the study representative. The sample consisted of 935 returned questionnaires compiled by an equal number of cat/dog owners from across Italy. A pilot version of the questionnaire had been presented to 100 people prior to conducting the survey in order to ascertain whether it was easy to understand.

### Structure of the questionnaire

The questions selected for the survey were based on those already present in the literature; in particular, those reported by Mascarello et al. [23]. Each question was developed with the assistance of experts (a veterinarian, a nutritionist and a marketing research specialist) in order to gather relevant information from owners about the target topics.

Ten questions, divided into two sections, were included in the survey. The first section, containing 9 multiple-choice questions, was designed to profile the population sample. The demographic variables included in the profiling were: gender, age, geographical area of residence (see Additional file 2), education, and occupation. Additional variables were included to profile the interviewees in terms of pet food purchasing attitudes (type(s) of animal owned, preferred marketing channel, type of purchased pet food, sources of pet nutrition information).

In the second part, the pet owners were asked to express their opinion using a 5-point scoring system (1 = not important at all, 2 = not very important, 3 = quite important, 4 = very important, and 5 = fundamental) on the relevance of 16 specific quality-associated characteristics in the decision-making process of choosing pet food. The surveyed characteristics included pet preferences, coat and stool appearance, food smell and presentation, label information, location of pet food facilities, ingredients, brand, price, recyclable packaging, and cruelty-free claim.

### Statistical analyses

The choice of the statistical analyses that were performed was made on the basis of the surveys designed by [23, 26]. The data generated were submitted to exploratory, correlation, and correspondence analyses. The exploratory analysis provided a description of the sample interviewed through frequency analysis, the use of synthetic indicators (median, mean, coefficient of variation), and the cross tabulation of specific variables in order to identify the main differences between the consumer groups. Bonferroni’s corrected Spearman Rho Correlation analyses were carried out in order to highlight highly or poorly connected features. A strong correlation was detected for a correlation coefficient R > ±0.5 and a weak correlation for a R < ±0.2. Finally, profiling of the respondents, according to the clusters of interest (age, education, occupation, geographical origin, type of pet food, dog and/or cat ownership) was achieved using a multivariate correspondence analysis approach between the scores and specific population segments. To this end, the data were first converted to Dummy variables, then grouped into specific Burt tables (one table for each target profiling feature), which were subsequently used for a multivariate correspondence analysis. When a specific preference class was poorly represented (less than 10 cases), the cases were assigned to the adjacent preference class. The relative weight of each class of preference was standardized by considering its percentage occurrence in each specific population segment. All the analyses were performed using PAST version 2.3 [27].

## Supplementary information

**Additional file 1.** Questionnaire: how is pet food quality assessed? In this file the questionnaire used during the survey is reported, translated in English language.

**Additional file 2.** Map of Italy. Map of Italy and segmentation in North, Center, South and Islands. Source: own source.

## Data Availability

The datasets analysed during this study are available from the corresponding author on reasonable request.

## Abbreviations

FEDIAF: European Pet Food Industry Federation
y: years
Reg. (EC) No.: Regulation of the European Community number
