# Supplementary material for: Profiling Italian cat and dog owners’ perceptions of pet food quality traits
Source: BMC Vet Res. 2020 May 11;16:131. doi: 10.1186/s12917-020-02357-9 (PMC7216655; doi:10.1186/s12917-020-02357-9)
Supplement: Supplementary file 1 — Additional file 1. Questionnaire: how is pet food quality assessed? In this file the questionnaire used during the survey is reported, translated in English language. [file 12917_2020_2357_MOESM1_ESM.pdf]

Università di Torino

Dipartimento di Scienze Veterinarie

Questionnaire: how is pet food quality assessed?

Part 1: Participant’s profile

Gender:
□ Female
□ Male

Age:
□ 18–34 years
□ 35–50 years
□ 51–64 years
□ More than 64 years

Geographical area:
□ North-West
□ North-East
□ Center
□ South and Islands

Education:
□ Primary / Secondary school
□ High school / Professional qualification
□ Degree / Postgraduate specialization

Occupation:
□ Student
□ Work at home
□ Retired
□ Worker
□ Other (specify)

Animal owned:
□ Dogs
□ Cats
□ Dogs and cats

Type of purchased pet food:
□ Dry food only (kibbles)
□ Wet food only (cans / trays)
□ Both kibble and cans

Preferred marketing channel (give only one answer):
□ Supermarket
□ Pet store
□ Online

Where do you find the information you need to make your purchase decision? (give only one answer)
□ Online – Blog
□ Online – Brand website
□ Veterinarian
□ Relatives and friends’ advice
□ Other (specify)

Part 2: Pet food characteristics seen as quality indicators

Score the following characteristics, indicating how important they are to you to ensure your pet food products a high level of quality.

Score from 1 to 5

1 = not important at all
2 = not very important
3 = quite important
4 = very important
5 = fundamental

Question: "To be a quality pet food product, it must…"

readily consumed by my dog/cat	**1**	**2**	**3**	**4**	**5**
give my dog / cat a nice coat	**1**	**2**	**3**	**4**	**5**
allow my dog / cat to produce normal stools	**1**	**2**	**3**	**4**	**5**
have a good smell	**1**	**2**	**3**	**4**	**5**
look good	**1**	**2**	**3**	**4**	**5**
clearly indicate where it is produced	**1**	**2**	**3**	**4**	**5**
be cruelty-free	**1**	**2**	**3**	**4**	**5**
label comprehension	**1**	**2**	**3**	**4**	**5**
contain natural ingredients	**1**	**2**	**3**	**4**	**5**
have meat at its main ingredients	**1**	**2**	**3**	**4**	**5**
contain fresh meat	**1**	**2**	**3**	**4**	**5**
have a high percentage of protein	**1**	**2**	**3**	**4**	**5**
not contains cereals	**1**	**2**	**3**	**4**	**5**
be of a well-known brand	**1**	**2**	**3**	**4**	**5**
cost more than the others	**1**	**2**	**3**	**4**	**5**
come in recyclable packaging	**1**	**2**	**3**	**4**	**5**
