# Supplementary figures and images for: Profiling Italian cat and dog owners’ perceptions of pet food quality traits
Source: BMC Vet Res. 2020 May 11;16:131. doi: 10.1186/s12917-020-02357-9 (PMC7216655; doi:10.1186/s12917-020-02357-9)

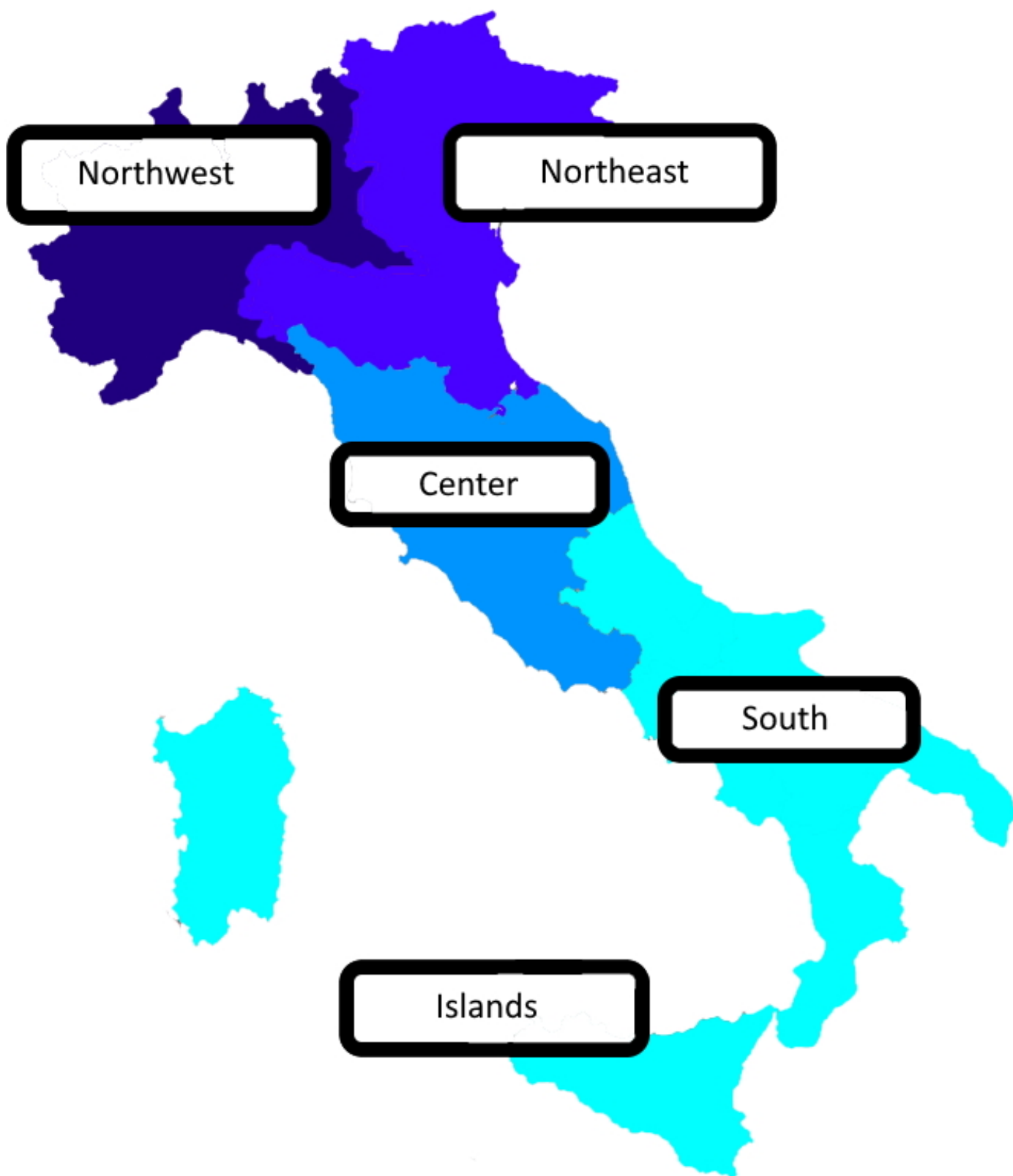

Northwest

Northeast

Center

South

Islands

Supplement: Supplementary file 2 — Additional file 2. Map of Italy. Map of Italy and segmentation in North, Center, South and Islands. Source: own source. [file 12917_2020_2357_MOESM2_ESM.pdf]
